# Risk of inflammatory bowel disease following a diagnosis of irritable bowel syndrome

**DOI:** 10.1186/1471-230X-12-55

**Published:** 2012-05-28

**Authors:** Chad K Porter, Brooks D Cash, Mark Pimentel, Akintunde Akinseye, Mark S Riddle

**Affiliations:** 1 Enteric Diseases Department, Infectious Disease Directorate, Naval Medical Research Center, 503 Robert Grant Avenue, Silver Spring, MD, USA; 2 National Naval Medical Center, Gastroenterology, Bethesda, MD, USA; 3 Cedars-Sinai Medical Center, Los Angeles, Gastrointestinal Motility Program and Laboratory, Los Angeles, CA, USA; 4 George Washington University, School of Public Health and Health Services, School of Medicine and Health Sciences, Washington, DC, USA

**Keywords:** Inflammatory bowel disease, Irritable bowel syndrome, Infectious gastroenteritis, Crohn’s disease, Ulcerative colitis

## Abstract

**Background:**

Irritable bowel syndrome (IBS) and inflammatory bowel disease (IBD) symptoms often overlap. In some IBS cases there are subtle inflammatory changes similar to the immune-mediated pathophysiology of IBD, and the risk of both increases after infectious gastroenteritis (IGE).

**Methods:**

To evaluate the effect of IBS and IGE on IBD risk utilizing US Department of Defense medical encounter data, active duty personnel with IBS were matched to subjects without IBS. Medical encounter history was analyzed to assess for incident IBD. IGE was identified from documented medical encounters and by self-report. Relative risks were calculated using Poisson regression models.

**Results:**

We identified 9,341 incident IBS cases and 18,678 matched non-IBS subjects and found an 8.6-fold higher incidence (*p* < 0.0001) of IBD among those with IBS (238.1 per 100,000 person-years) compared to our referent population (27.8 per 100,000 person-years). In a subset (n = 2,205) of well-defined IBS cases, IBD risk was 15 times that of subjects without IBS. The median time between IBS and IBD diagnoses was 2.1 years. IGE also increased IBD risk approximately 2-fold ( *p* < 0.05) after controlling for IBS.

**Conclusions:**

These data reflect a complex interaction between illness presentation and diagnosis of IBS and IBD and suggest intercurrent IGE may increase IBD risk in IBS patients. Additional studies are needed to determine whether IBS lies on the causal pathway for IBD or whether the two are on a pathophysiological spectrum of the same clinical illness. These data suggest consideration of risk reduction interventions for IGE among IBS patients at high disease risk.

## Background

Functional gastrointestinal disorders (FGD) are common and cause considerable morbidity. One of the most common FGD, irritable bowel syndrome (IBS), affects approximately 12% of the global population and results in over $30 billion in direct and indirect medical costs annually in the US [1-3]. A less common, yet more severe group of disorders that shares many of the clinical symptoms of IBS are inflammatory bowel diseases (IBD), often requiring immunosuppression or surgery to control symptoms and morbid disease processes. Despite differences in clinical morbidity and pathophysiology, given the considerable overlap of symptoms, some have argued that IBS and IBD may represent clinical manifestations of a pathophysiologic spectrum of disease [4]. The hypothesis is that subclinical inflammation and immune activation resulting from long-term IBS precede the expression of IBD. Two relatively large population-based studies have shown a significant increase (5–10 fold) in the risk of IBD among those with IBS compared to those with no prior IBS history, with some indication that the effect may be greater for Crohn’s disease [5,6]. However, similarities in symptom presentation between the two conditions could lead to misdiagnoses of less severe IBD and confound this observation [7,8].

Recent research also points to a potential increased IBD risk following acute infectious gastroenteritis (IGE). In a large European cohort study, prior IGE had a 2.4- fold (95% CI, 1.7–3.3) increased risk of IBD compared to subjects with no IGE, with the highest risk in the first year after the IGE episode [5]^,^. In an additional study we found prior IGE significantly increased the risk of IBD (OR 1.53, 95% CI 1.4–1.7) after controlling for important covariates including prior IBS diagnosis [6]. More recent studies have found increased IBD risk after Campylobacter or Salmonella infections [9,10].

Due to the well-described association between IGE and IBS [11,12] and the potential association between IGE, IBS, and IBD, we conducted a retrospective cohort study on a healthy population with open access to health care and electronic medical record data to evaluate the differential risk of IBD among those with and without IBS, the risk of IGE in subjects with IBS, and the effect of an intercurrent IGE episode on subsequent IBD risk.

## Methods

This was a retrospective cohort study utilizing subjects identified from the Defense Medical Surveillance System (DMSS) from 1998 – 2008. Medical data were obtained from ambulatory and inpatient claims data for care obtained within the Military Health Services and the Tri-Service Reportable Events System databases. Demographic information was obtained from personnel records and deployment data were derived from deployment rosters and post-deployment health assessments. Selection of subjects was limited to Active Duty personnel.

The primary risk factor was new-onset IBS identified by the first medical encounter in which an ICD-9 code of 564.1 was given (in any diagnostic position). IBS was stratified into two sub-categories, “IBS” and “well-defined IBS”. For “IBS”, subjects had a minimum of two separate medical encounters with an ICD-9 code of IBS recorded. For “well-defined IBS”, subjects had an initial medical encounter with an IBS diagnosis, a concurrent and/or subsequent medical encounter (no greater than 12 months) in which there was a endoscopic procedural code to include diagnostic sigmoidoscopy and/or diagnostic colonoscopy and a subsequent medical encounter (no greater than 12 months) with an additional ICD-9 code of IBS and no intercurrent diagnoses of IBD. Matched non-IBS subjects were randomly selected from persons with unrelated acute or chronic diseases within 1 year of the identified IBS subject at the same military treatment facility (MTF) and in the same clinical setting (inpatient/outpatient). Subjects meeting the IBD diagnosis within 1 year of entrance into the observational period were excluded to minimize potential IBS disease misclassification.

The primary outcome was IBD, categorized into two disease phenotypes: ulcerative colitis (UC) and Crohn’s disease (CD). IBD subjects were classified as someone with an ICD-9 code of 555.0, 555.1, 555.9, and 556 (all subgroup codes). Analyses evaluated UC and CD together and separately. A diagnosis of pseudopolyposis colon (556.4) was also included in the overall IBD analyses, but not included in the analyses of UC and CD separately as it can be typified as either disease sub-type. Subjects must have been diagnosed with the same condition on two separate medical encounters to be counted as having that outcome. Subjects meeting both case definitions (CD and UC) were classified as indeterminate colitis and were included in analyses of CD and UC.

Incidence rates were calculated using the number of incident IBD cases and total observed person time and were adjusted to a standard reference (per 100,000 years). Ninety-five percent confidence intervals (95% CI) were also calculated using a Poisson distribution. Subjects were censored at the time of leaving active duty service or at the first medical encounter in which a subsequently confirmed IBD diagnosis was recorded.

The primary covariate evaluated was IGE, determined by ICD-9 codes for bacterial and viral pathogens according to four main diagnostic groupings: pathogen specific, pathogen not specified, protozoan and viral codes. Other covariates evaluated included age, sex, military rank, educational status, marital status, branch of service, place of deployments, and duration of deployments.

Poisson regression models were developed using a backwards elimination approach to evaluate the relationship between IBS and IBD. Only variables significant at an *alpha* = 0.20 were retained in the final models. The model developed from all IBD was applied to each IBD phenotype. Similar methodology was used to assess the relative risk of IGE following a diagnosis of IBS. Statistical analyses were performed using SAS v. 8.2 for Windows (SAS Institute, Cary, NC). Two-tailed statistical significance was evaluated using an *alpha* of 0.05.

The study protocol was approved by the Naval Medical Research Center Institutional Review Board in compliance with all applicable Federal regulations governing the protection of human subjects.

## Results

**Figure 1:**
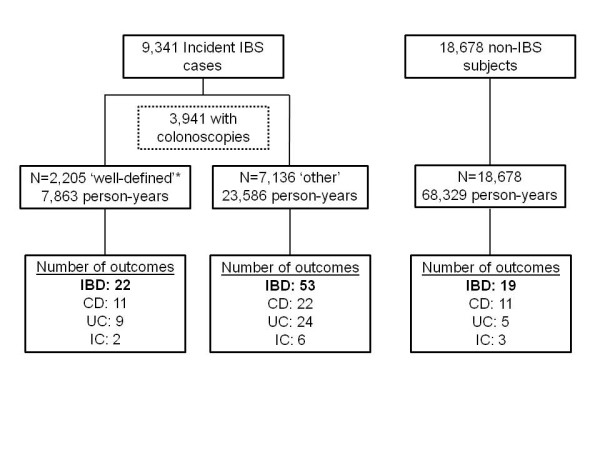
**Flow diagram of study population.** IBS: irritable bowel syndrome; IBD: inflammatory bowel disease; CD: Crohn’s disease; UC: ulcerative colitis; IC: indeterminate colitis. * Well-defined IBS: initial IBS diagnosis, a subsequent medical encounter (≤12 months) with sigmoidoscopy and/or diagnostic colonoscopy and a subsequent medical encounter (≤ 12 months) with an additional ICD-9 code of IBS with no IBD medical encounters.

A total of 9,341 IBS patients were included in the study and followed for incident IBD (Figure 1). Of those, 42.2% (n = 3,941) underwent a colonoscopy during the surveillance period with a median time of 51 (IQR: 14, 200) days between the first IBS diagnosis and initial colonoscopy. Just over 55% (n = 2,205) of those IBS patients had a subsequent IBS-related medical encounter within 1 year of the colonoscopy (without meeting the IBD case definition) and subsequently met the well-defined IBS definition. A total of 18,678 non-IBS subjects were included in the referent cohort. The non-IBS subjects were identified from 200 ICD-9 codes and included subjects with arthropathies and related disorders (ICD-9: 719; n = 1,760; 9.4%), unspecified disorders of the back (ICD-9: 724; n = 4,985; 26.7%), hypertension (ICD-9: 401; n = 1,756; 9.4%) and disorders of refraction and accommodation (ICD-9: 367; n = 2,891; 15.5%). The average age of subjects with IBS was 30.4 (standard deviation {SD}: 8.0) slightly older than the non-IBS referent cohort (mean age: 28.5; SD: 7.9; *p* < 0.001). In general, the demographics of the study population were representative of the active duty military population in terms of race, education, branch of service, rank and marital status (Table 1). However, females were more commonly represented in the IBS cohorts than in the non-IBS cohort (43.5% and 19.6%, respectively; p < 0.001). The average follow-up duration was 3.6 years (SD: 2.8), totaling 99,846 person-years of observation.

**Table 1:** Characteristics of cohort

**Variables**	**Well-defined IBS cohort**	**Other IBS cohort**	**Non-IBS cohort**
**N**	**2,205**	**7,136**	**18,678**
**Mean (SD) age[1]**	31.2 (7.8)	30.1 (8.0)	28.5 (7.9)
**N (%) male**	1281 (58.1)	3996 (56.0)	15014 (80.4)[2]
**Race [N (%)]**			
Black	394 (17.9)	1238 (17.4)	3397 (18.2)
White	1492 (67.7)	4792 (67.2)	11894 (63.7)
Other	319 (14.5)	1106 (15.5)	3387 (18.1)
**Education level [N (%)][1]**			
High school	1348 (61.1)	4514 (63.3)	12721 (68.1)
At least some college	568 (25.8)	1771 (24.8)	3772 (20.2)
Masters or doctorate	186 (8.4)	505 (7.1)	1182(6.3)
Unknown	103 (4.7)	346 (4.9)	1003 (5.4)
**Service [N (%)][1]**			
Army	736 (33.4)	2328 (32.6)	6137 (32.9)
Air Force	783 (35.5)	2476 (34.7)	6080 (32.6)
Navy	510 (23.1)	1649 (23.1)	4300 (23.0)
Marines	124 (5.6)	467 (6.5)	1573 (8.4)
Coast Guard	52 (2.4)	216 (3.0)	588 (3.2)
**Rank [N (%)][1]**			
Enlisted	1813 (82.2)	5935 (83.2)	15704 (84.1)
Officer/Warrant officer	392 (17.8)	1201 (16.8)	2974 (15.9)
**Marital status [N (%)][1]**			
Married	1358 (61.6)	4153 (58.2)	9740 (52.2)
Single	668 (30.3)	2514 (35.2)	8228 (44.1)
Other	174 (7.9)	461 (6.5)	680 (3.6)
Unknown	4 (0.2)	8 (0.1)	30 (0.2)
**N (%) deployed during surveillance period**	554 (25.1)	1878 (26.3)	2432 (26.0)
**Primary outcomes**			
N (%) with IBD	22 (1.0)	53 (0.7)	19 (0.1)
N (%) with UC	11 (0.5)	22 (0.3)	11 (0.06)
N (%) with CD	9 (0.4)	24 (0.3)	5 (0.03)
N (%) with IC	2 (0.09)	6 (0.08)	3 (0.02)
**Mean (SD) years of follow-up**	3.6 (2.7)	3.3 (2.7)	3.7 (2.8)

[1] At start of follow-up period.

[2] 2 subjects with missing data.

For those with IBD (n = 94), a diagnosis of UC was slightly more common than CD (58.5% and 52.1%, respectively) and 11 (11.7%) cases received multiple diagnoses of both IBD subtypes. The incidence of IBD was 94.1 per 100,000 person-years (95% CI: 76.9, 115.2). However, this rate was approximately 8-fold higher in those with a prior IBS diagnosis than in those with no prior IBS, 238.1 per 100,000 person-years (95% CI: 189.8, 298.5) and 27.8 per 100,000 person-years (95% CI: 17.7, 43.6), respectively (*p* < 0.0001). Limiting the analysis to those with well-defined IBS, the IBD incidence rate increased to 279.2 cases per 100,000 person-years (95% CI: 183.9, 424.1). In a multivariate analysis (Table 2), the increased risk for IBD among those with antecedent IBS compared to those with no prior IBS remained (RR: 9.42; 95% CI: 5.65, 15.70). Male gender (RR: 2.16; 95% CI: 1.32, 3.52) and intercedent IGE medical encounters during the surveillance period (RR: 2.19; 95% CI: 1.01, 4.75) were also independently associated with increased IBD risk. In contrast, deployment during the surveillance period was associated with a decreased risk of IBD (RR: 0.34; 95% CI: 0.12, 0.92).

**Table 2:** Incidence and crude and adjusted rate ratios of inflammatory bowel disease in a retrospective cohort study of active duty US military personnel from 1998 to 2008

	**All IBD**			**CD**			**UC**		
	Incidence*	**cRR (95% CI)**	**aRR (95% CI)**	Incidence*	**cRR (95% CI)**	**aRR (95% CI)**	Incidence*	**cRR (95% CI)**	**aRR (95% CI)**
**Female**	81.5	1.0	1.0	23.3	1.0	1.0	46.6	1.0	1.0
**Male**	98.5	1.21 (0.74, 1.96)	2.21 (1.35, 3.62)	43.2	1.85 (0.78, 4.44)	3.55 (1.47, 8.54)	43.2	0.93 (0.48, 1.80)	1.66 (0.84, 3.28)
**≤1 IGE**	89.4	1.0	1.0	40.1	1.0	1.0	37.0	1.0	1.0
**>1 IGE**	274.7	3.07 (1.42, 6.64)	2.19 (1.01, 4.75)	196.2	4.90 (1.93, 12.42)	1.51 (0.36, 6.32)	78.5	2.12 (0.51, 8.81)	3.49 (1.36, 8.95)
**Not deployed**	104.3	1.0	1.0	40.5	1.0	1.0	49.8	1.0	1.0
**Deployed**	29.6	0.28 (0.10, 0.77)	0.33 (0.12, 0.91)	22.2	0.55 (0.17, 1.78)	0.65 (0.20, 2.13)	7.4	0.15 (0.02, 1.08)	0.17 (0.02, 1.27)
**No IBS**	27.8	1.0	1.0	7.3	1.0	1.0	16.1	1.0	1.0
**IBS**	238.1	8.56 (5.18, 14.17)	9.40 (5.64, 15.6)	104.7	14.32 (5.59, 36.67)	17.30 (6.71, 44.62)	104.7	6.51 (3.29, 12.88)	6.53 (3.25, 13.11)

* Per 100,000 person-years.

IBD: inflammatory bowel disease; CD: Crohn’s disease; UC: ulcerative colitis; cRR: crude rate ratio; aRR: adjusted rate ratio; CI: confidence interval.

Estimates of CD and UC incidence were quite similar (38.1 and 44.1 per 100,000 person-years, respectively). When analyzing IBD subtypes in the same model established for all IBD, the relative risk of CD among those with IBS compared to those without IBS was approximately twice the UC relative risk {14.32 (95% CI: 5.59, 36.67) and 6.51 (95% CI: 3.29, 12.88), respectively}, although not statistically different. Similarly, when limiting analyses to the well-defined IBS cohort, the relative risk of CD was 1.6-fold higher in those with IBS compared to those without (CD: 18.4; *p* = 0.006; UC: 11.2; *p* = 0.002). No other rate ratio estimates were significantly different when the non-IBS cohort was stratified by acute or chronic illnesses matching (data not shown).

**Figure 2:**
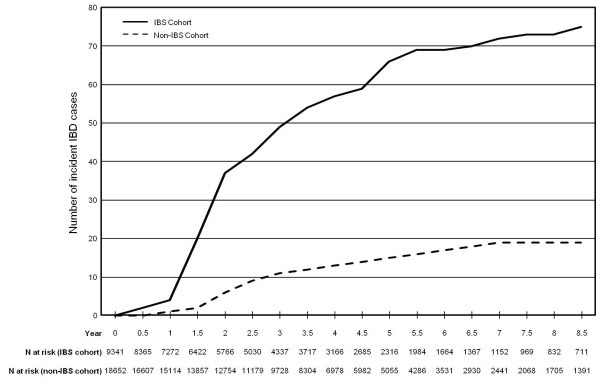
Number of incident IBD cases over time in active duty US military personnel.

Incident IBD diagnoses by antecedent IBS diagnosis are shown in Figure 2. For subjects with IBS who were subsequently diagnosed with IBD, the median time to the first IBD diagnosis was 2.1 years (IQR: 1.4, 3.8). There was no significant difference (*p* = 0.5) in the time to reaching an IBD outcome for subjects with UC (median: 2.1 years; IQR: 1.4, 4.4) or CD (median: 1.8 years; IQR: 1.4, 3.3). While the time to meeting the IBD outcome definition was more prolonged for subjects without an antecedent IBS diagnosis (median: 2.6; IQR: 1.9, 4.5), this difference was not statistically significant ( *p* = 0.2). Of the subjects with antecedent IBS who ultimately met the IBD definition, 28 (37.3%) continued to be coded for IBS for a median 278 (IQR: 66, 698) days after IBD diagnosis.

IGE during the follow-up period was associated with an increased risk of IBD after controlling for IBS, deployment and gender (Table 2). Additionally, during the follow-up period the incidence of medical encounters for IGE in the IBS cohort was 2.4-fold that of the non-IBS cohort (*p* < 0.001) (Table 3). This effect was apparent for all IGE infection diagnosis groups (e.g. bacterial, viral, and protozoal). Limiting analyses to a subset of subjects who were deployed to Iraq or Afghanistan in 2008, compared to subjects with no IBS, those with IBS were almost 3 times as likely (RR: 2.7; 95% CI: 1.8, 4.0) to self-report a diarrheal episode that prevented the subject from performing their duties (*p* < 0.001). Among those with IBS who reported diarrhea during deployment 39.6% reported ongoing diarrheal symptoms after the deployment. This was significantly higher ( *p* < 0.001) than in subjects with IBS who did not self-report diarrhea during deployment (7.1%). Similarly, among subjects without diagnosed IBS, those reporting an acute diarrheal episode during deployment were more likely ( *p* < 0.001) to report ongoing diarrheal symptoms after deployment (11.5%) than were those with no reported acute diarrhea episode during deployment (2.7%).

**Table 3:** Relative incidence and risk of infectious gastroenteritis-associated medical encounters by antecedent IBS diagnosis

	**IBS cohort**		**Non-IBS cohort**		**RR (95% CI)**
	**Number of episodes**	Incidence*	**Number of episodes**	Incidence*	
**All IGE**	1088	49.1	1083	20.2	2.4 (2.3, 2.6)
**Bacterial IGE**	600	19.0	449	6.6	2.9 (2.6, 3.3)
**Viral IGE**	915	29.0	925	13.5	2.1 (2.0, 2.4)
**Protozoal IGE**	23	1.0	9	0.1	7.7 (3.7, 16.2)

*Incidence per 1,000 person years.

## Discussion

We found the incidence of IBD in the cohort of persons with a prior diagnosis of IBS was approximately 9 times higher than in the referent cohort (non-IBS). Similarly, Garcia-Rodriguez et al. found a 16.3-fold increased risk of IBD in a cohort of patients with IBS, compared to the general population, an association that was more pronounced for CD than UC [5]. Using a case–control design, we previously reported on this association in a comparable population, albeit at a lower magnitude than reported by Garcia-Rodriguez et al. [6]. This retrospective cohort study builds on these two initial reports using a more well-defined study population and detailed evaluation of medical encounters for IBS and non-IBS subjects. Additionally, we found that the rate ratio associated with IBS was higher for CD than UC, also consistent with the prior studies.

The association between IBS and IBD is well-recognized; however, the mechanism(s) underlying this association is a source of ongoing research. Some have argued that IBS and IBD represent clinical presentations on a pathophysiologic spectrum of disease given the considerable overlap between symptoms in patients with IBS and IBD, whereby IBS symptoms represent sub-clinical inflammation and immune activation that progress in severity towards the expression of IBD [4,13]. Increased mucosal barrier defects in some patients with IBS may also contribute to the increase passage of luminal antigens of dietary and bacterial origin into the sub-mucosal which may result in the further activation of mucosal immune responses involved in the genesis of IBD [14].

Another common link between these two disorders is the independent associations with antecedent *Campylobacter jejuni* infection which have been described [9,10,15-17]. *C. jejuni*, a leading cause of enterocolitis worldwide, has been shown to permit the translocation of normal, noninvasive microflora via novel processes that implicate epithelial lipid rafts and M-cell transport and induce a proinflammatory response [18-20]. This disruption in intestinal barrier function may prime the intestine for chronic inflammatory responses in susceptible individuals. Follow-on genetic studies of the Walkerton, Ontario STEC-Campylobacter outbreak found that subjects with single nucleotide polymorphisms for genes encoding proteins involved in epithelial cell barrier function and the innate immune response to enteric bacteria (TLR9, IL6, and CDH1) were independently associated with development of IBS following acute gastroenteritis [21]. Interestingly, recent experimental and clinical evidence implicate aberrant CDH1 function related to maintenance of epithelial barrier integrity with increased risk of IBD as well [22,23]. Furthermore, Chae *et al.* reported that genotype and allelic frequencies (among four single nucletide polymorphisms) of TNFRSF17, a gene expressed in mature B cells and thought to be important for B cell development and autoimmune response, were similar in IBS and UC patients compared to controls [24]. Finally, recent findings have suggested that there may be similarities in serotonin signaling between IBS and UC patients that could explain the altered motility, secretion, and sensation common to both [25]. Clearly, more studies are needed to evaluate the potential overlap in pathogenesis between these two disorders in terms of genetic, host and environmental interactions which may support the observed epidemiological findings.

Another potential explanation for the observed association may be the symptom overlap between IBS and IBD patients and possible misdiagnoses of IBS in patients with IBD. In an effort to minimize this effect, we analyzed a subset of our IBS cohort that had a colonoscopy without an IBD diagnosis while receiving IBS-related medical care. In this subset analysis, IBD incidence remained significantly elevated compared to the non-IBS subjects (RR: 15.0; 95% CI: 4.5, 50.1). IBS is often considered a diagnosis of exclusion, and while we assume that our well-defined IBS definition includes the result of a “negative colonoscopy”, such an assumption does not rule out potential microscopic colitis which is associated with similar symptoms and normal endoscopic mucosal appearance on colonoscopy and may not have been diagnosed [26,27]. The utilization of our well-defined IBS population may have resulted in a biased selection of IBS patient types, perhaps those who had more severe disease with underlying inflammatory/immune dysfunction not detected on colonoscopy or histopathology or those more likely to have a diarrheal component. In fact, in a number of patients with IBS, the colonic mucosa appears normal; however, there are histopathological changes in the rectum, a region not commonly biopsied during colonoscopy for IBS symptoms. Furthermore, it is possible that IBS subjects may have had IBD, specifically, Crohn’s disease, for which small intestine mucosal abnormalities were not visualized. In our data, the relative risk of CD in those with well-defined IBS was 18.4 compared to the non-IBS comparator group. This was slightly higher than the estimates utilizing all IBS subjects (RR: 11.2) which supports misclassification may be present. However, our findings of increased risk for UC for which colonoscopic and/or histopathological diagnosis are likely more sensitive, suggest that in “colonoscopy negative” IBS patients there is an increased risk of developing UC. In total, these data suggest that IBS patients are at increased risk of IBD and additional studies are needed to ascertain whether certain genetic or immunologic abnormalities underlie both of these conditions, and determine which subset of IBS patients may be at higher IBD risk.

We found a median IBD onset time of 2.1 years (IQR: 1.4, 3.8) with no significant difference between UC and CD (2.8 and 2.1 years, respectively). Furthermore, there was no difference in the time to IBD onset for subjects with no prior history of IBS (median: 2.6 years; IQR: 1.9, 4.5). In contrast, Pimentel *et al.* noted a prodromal period for patients who were ultimately diagnosed with UC or CD with a longer delay in CD patients (7.7 years) than UC patients (1.2 years) [7]. A possible explanation for the apparent discrepancy in prodromal periods between the two studies may be the method by which these data were obtained or differences in healthcare access or health-seeking behavior between study populations. While the study by Pimentel *et al.* used a self-reported questionnaire of subjects after IBD onset, our study utilized medical encounter data. Both methodologies have inherent limitations and potential sources of bias.

We also found a 4-fold increased risk of IBD among subjects with more than 1 documented episode of IGE after controlling for other important covariates. Others have reported similar associations between antecedent IGE and IBD^,^[6,9,10,16]. The consistency of this finding seems to point to a direct association, though a causal link has not yet been established. Other covariates were associated with differential IBD risk. Specifically, males had a slightly higher risk of IBD than females. However, looking at IBD subtypes, this association was only significant with CD. In contrast, a 1999 population-based study of IBD incidence in a Canadian province showed a significantly higher rate of CD in women than in men, and a similar rate of UC in both genders [28]. A 2008 report on IBD incidence in participants in a specific managed care organization found similar rates of UC and CD among men and women [28]. One possible explanation is that the population based study did not control for comorbid IBS, a FGD that is known to be more common in females [29]. Deployment during the study period was associated with a decreased IBD risk. At first glimpse, this appears contradictory to what may be expected given that deployments are frequently to regions at high risk of IGE, often associated with bacterial pathogens linked to an increased IBD risk [9,10]. However, the likely explanation of this inverse association is that of a healthy worker effect. As stated previously, there is a recognized prodrome for IBD. It is reasonable to assume that subjects with such a complex of symptoms may be less likely to be deployed due to undiagnosed health concerns precluding one from adequately performing in a deployed setting. A similar finding has been reported with non-specific arthralgia and arthropathy [30]. Alternatively, there may be an inherent delay in the diagnosis of IBD in military populations, as this and similar diagnoses can result in medical discharge, [6] and military personnel who are motivated to deploy may be of a type that are less likely to seek care for chronic underlying diseases in order to avoid diagnosis and separation.

In addition to the association between IBS and IBD, we found an increased incidence of IGE among our IBS cohort, compared to subjects without IBS. While the risk of IBS following IGE, termed post-infectious IBS (PI-IBS), has been well documented [11], to our knowledge, this is the first report of increased IGE risk in subjects with IBS. DuPont et al. reported a worsening of functional GI symptoms following an episode of travelers’ diarrhea (TD) in subjects with IBS [31]. However, the authors did not assess whether the risk of TD was higher among those with IBS. We were limited in our assessment of infectious gastroenteritis (IGE) to only episodes associated with a medical encounter, and it has been well-established that only a small proportion of IGE episodes actually seek medical care [32]. Therefore, it is possible that the observation of an increased rate of IGE in the IBS cohort may be related to the fact that those subjects were more likely to seek care for an incident IGE episode than their non-IBS counterparts. Thus, this observation may be solely due to differences in care seeking behavior or differences in the severity of diarrheal and non-diarrheal symptoms such as abdominal pain or cramps, nausea, malaise or myalgia. In an effort to evaluate differences in the care seeking behavior in our 2 study populations, we analyzed the number of inpatient and outpatient medical encounters and found that after removing IBS- and IBD-associated visits, IBS subjects were more likely (*p* < 0.001) to present for medical care (median number of outpatient visits: 49; IQR: 20, 89) than were the non-IBS comparator cohort (median: 29; IQR: 14, 56). An alternative explanation is that due to the common clinical features, IBS symptoms may have been misdiagnosed as being of infectious etiology. Unfortunately, inadequate sample collection and microbiology is commonplace with infectious gastroenteritis [32], so we are unable to assess this potential bias.

The mechanism(s) by which IBS may increase one’s susceptibility to specific pathogens are unknown; however, there are several potential possibilities. First, in patients with IBS, there is a modification of genetic expression and secretion of important chemokines such as interleukin 8 (IL-8), which is decreased in those with IBS [33]. IL-8 is also known to recruit neutrophils to the intestinal mucosa during infection with organisms associated with travelers’ diarrhea [34]. Another possibility is that the modified intestinal microbiome in a subset of IBS patients may influence subsequent IGE risk [35]. Furthermore, in a subset of IBS patients evidence of mucosal barrier dysfunction is apparent which could increase susceptibility to enteric infection [36-38]. Of note, we found a significantly higher incidence of protozoal-attributed IGE than was seen for either bacterial or viral-associated IGE though the lack of pathogen-specific data in this study precludes an assessment of pathogen-specific risks. These findings need validation in a different population, and if found to be consistent would have certain implications on considerations regarding management of patients with IBS in situations where IGE risk is high. Travelers from developed to developing countries and deployed military personnel have consistently high attack rates of travelers’ diarrhea [39,40]. Further study is needed to evaluate whether certain IBS subtypes, including PI-IBS, are at increased risk for IGE and IBD. If the association between IBS, IGE and IBD is found in additional studies of other populations, increased emphasis on preventive efforts may be needed in those subpopulations with IBS to include consideration of antibiotic chemoprophylaxis as currently recommend for high-risk groups [41,42].

As previously stated, there are inherent limitations to the data presented herein and the results should be interpreted with caution. First, the use of a medical encounter database is a potential source for misclassifications of exposure, outcome and other covariates due to inaccurate ICD-9 or CPT coding. We attempted to reduce the impact of this misclassification by requiring multiple medical encounters to document both the exposure and outcome of interest as has been described previously [43]. Incident IBS was defined as the first medical encounter for which a diagnosis of IBS was given to a case that subsequently went on to meet the IBS case definition. Certainly utilization of the first IBS-related medical encounter as ‘new onset’ may not account for subjects with a pre-existing diagnosis of IBS prior to his/her military service which may have artificially decreased the diagnosis time. Importantly, as part of the military screening process, potential servicemembers are screened to ensure they are in good general health. Screening for functional bowel disorders is not done, nor would it be exclusionary. Thus we cannot necessarily rule out someone with pre-existing IBS; however, it does minimize the potential impact of this limitation inherent in this study design. Additionally, we analyzed a subset of subjects for which colonoscopies had been performed in an attempt to remove potential misclassification of our exposure of interest. Unfortunately, due to a lack of specific ICD-9 codes, we were unable to analyze IBS subtypes and their potential differential effect on IBD risk. Another limitation inherent with these data is our inability to capture other important covariates, such as smoking and stress. We also were unable to completely account for subjects with pre-existing IBS, artificially decreasing the diagnosis time from IBS to IBD. Future studies utilizing more prospective designs should evaluate the potential differential effect these covariates may have on the reported associations. Additionally, while we noted an increase in the relative risk of IBD associated with IBS, the absolute risk of IBD was low and limitations in ICD-9 codes may have contributed to observed associations.

## Conclusions

In summary, we found a significant increased risk of IBD among a cohort of subjects with IBS compared to a matched reference cohort with no prior IBS. Additionally, we found that intercurrent infectious gastroenteritis further increased this IBD risk. Future studies in other populations using well controlled prospective study designs are needed to verify these results, though would be challenging due to the large numbers of subjects that would need to be followed. However, these data reflect the complex interaction between functional and inflammatory bowel disorders and implicate a common pathogenesis and important role of infectious gastroenteritis.

## Competing interests

The authors declare that they have no competing interests.

## Authors’ contributions

CP, MR: study concept & design. CP, MR, AA, BC and MP: analysis, interpretation and drafting manuscript. All were involved in critical revision of manuscript. All authors read and approved the final manuscript.

## Copyright statement

Authors are employees of the U.S. Government and military service members. This work was prepared as part of official duties. Title 17 U.S.C. §105 provides that ‘Copyright protection under this title is not available for any work of the United States Government.’ Title 17 U.S.C. §101 defines a U.S. Government work as a work prepared by a military service member or employee of the U.S. Government as part of that person’s official duties.

## Disclaimer

The views expressed in this article are those of the authors and do not necessarily reflect the official policy or position of the Department of the Navy, Department of Defense, nor the U.S. Government. This is a US Government work. There are no restrictions on its use. There were no financial conflicts of interests among any of the authors. This study was conducted under support of the Military Infectious Disease Research Program and Department of Defense Global Emerging Infections Surveillance and Response System funding.

## Pre-publication history

The pre-publication history for this paper can be accessed here:

http://www.biomedcentral.com/1471-230X/12/55/prepub
